# Fertility protection in the reproductive-age population: A evolutionary concept analysis

**DOI:** 10.1016/j.ijnss.2026.04.011

**Published:** 2026-04-22

**Authors:** Yaxin Chen, Xin Qi, Mengyuan Yang, Jing Li, Lei Wu, Yaya Zhang, Zhentao Yang, Ruirui Wang, Yanhong Wang

**Affiliations:** School of Nursing, Lanzhou University, Lanzhou, China

**Keywords:** Concept analysis, Definition, Evolutionary method, Fertility protection, Reproductive-age population

## Abstract

**Objectives:**

This study aimed to report a concept analysis of fertility protection and elucidate a current definition.

**Methods:**

This study adopted Rodgers’s evolutionary and inductive method of concept analysis. Literature from January 1990 to March 2025 was searched using PubMed, Web of Science, China National Knowledge Infrastructure (CNKI), and the China Biomedical Literature Database (CBM). Data extraction involved identifying surrogate and related terms, attributes, antecedents, and consequences of fertility protection. The extracted data were analyzed using thematic content analysis.

**Results:**

Based on a review of 40 studies on fertility protection, the attributes include preventive interventions, promoting the release of fertility potential, and an orientation towards building a birth-friendly society. The antecedents were classified into six categories: physiological factors, pathological factors, iatrogenic factors, psychological factors, environmental and lifestyle factors, and social factors. The consequences were systematically categorized into three domains: individual, familial, and societal. Fertility protection was defined as the maintenance or preservation of an individual’s fertility based on preventive interventions, while at the same time promoting the full exertion of their fertility potential through a combination of medical technology, policy, economic, and socio-cultural means, thereby achieving the ultimate goal of a birth-friendly society.

**Conclusions:**

Fertility protection is a complex field involving multiple disciplines. This study clarifies the conceptual attributes of fertility protection through concept analysis. It provides a common reference point to promote effective collaboration and communication among nursing, reproductive medicine, public health, and policy research.

## What is known?

• Fertility protection has received increasing attention in reproductive medicine, oncology, and public health as an important strategy for maintaining fertility in individuals exposed to fertility-threatening factors.
• Existing literature has mainly addressed fertility protection within disease-specific contexts or as fertility preservation technologies, while its conceptual boundaries and core components remain insufficiently defined.

## What is new?

• This study shows that fertility protection is not limited to preserving reproductive function or preventing fertility loss, but also includes enabling the realization of fertility potential.
• It further reveals that fertility protection extends beyond individual-level biomedical intervention and should be understood as a broader life-course strategy supported by medical, psychosocial, policy, economic, and socio-cultural factors, with the ultimate orientation toward a birth-friendly society.
• This study reframes fertility protection from a narrow biomedical intervention to a broader life-course and birth-friendly social strategy.

## 1 Introduction

A stable and sustainable population is fundamental to national economic and social development. The total fertility rate (TFR) is an internationally recognized rational metric for the sustainability of population, the economy, and society. According to data published in China’s Seventh Population Census [1], the TFR for women of childbearing age in 2020 was 1.3, which is extremely low. The declining fertility of people of childbearing age is an important factor contributing to the low fertility rate. Data surveys show that the global prevalence of infertility has climbed from 11.0 % in 1990 to 17.5 % in 2021, and the prevalence of infertility in China is 18.2 % [2,3]. This rise is closely associated with late marriage and childbearing [4,5], environmental pollution [6], and lifestyle changes [7]. Declining fertility has become a major social challenge, affecting not only the well-being of individual families but also demographic structure and broader social development [8,9]. Against this background, fertility protection has become increasingly important. Biologically, fertility refers to the maximum number of children a healthy man and woman can have without birth control [10]. Sociologically, it refers to the maximum number of children that can be supported under existing economic and social conditions [11]. Thus, while natural fertility sets the upper biological limit, social factors such as attitudes toward marriage and parenthood, fertility preferences, economic pressure, family relationships, mental health, and quality of life influence whether this potential is translated into actual fertility [12]. Fertility protection is aimed at maintaining and improving individual fertility through a series of medical, technological, and social measures to prevent and reduce the damage to fertility caused by various factors [13]. It is therefore both a fundamental strategy for preserving baseline fertility and an important approach to enhancing fertility.

Despite growing attention to fertility protection in clinical practice and public health, the concept remains underdeveloped. Existing studies have mainly discussed fertility protection within specific clinical contexts, technological applications, or adjacent fields such as fertility preservation and reproductive health [13,14]. At the same time, few studies have systematically examined its conceptual structure. As the scope of fertility protection has expanded from a narrowly biomedical focus to a broader life-course and biopsychosocial perspective, its defining attributes, antecedents, and consequences have become increasingly complex but remain inadequately clarified. This lack of conceptual clarity limits consistent use of the term and weakens its applicability in research, clinical practice, health education, and policy development. Therefore, a concept analysis is needed to provide a clearer theoretical foundation for understanding and applying fertility protection.

Rodgers’s evolutionary approach conceptualizes terms as dynamic, context-sensitive, and shaped by temporal, disciplinary, and social influences, making it particularly suitable for analyzing fertility protection [15]. Therefore, this study adopted Rodgers’s evolutionary approach to conduct the concept analysis of fertility protection.

## 2 Methods

### 2.1 Rodgers’s evolutionary method

Rodgers’s method includes six steps that are listed sequentially, but they are actually developed iteratively: 1) identifying the concept of interest and related terms; 2) selecting the appropriate literature, setting, sample, and data sources; 3) collecting data relevant to the concept, including its related terms, development, attributes, antecedents, and consequences; 4) analyzing and synthesizing the data to clarify the characteristics and development of the concept; 5) identifying a model case to illustrate the concept; and 6) identifying implications for further development of the concept.

### 2.2 Search strategy and study selection

The databases searched for relevant articles included PubMed, Web of Science, China National Knowledge Infrastructure (CNKI), and China Biomedical Literature Database (CBM). The databases were searched using the “OR” operators in the titles, abstracts, and keywords, and search keywords of “fertility protection” OR (“fertility preservation” NOT “cryopreservation”) OR “impaired fertility” OR “decreased fertility” OR “reduced fertility”. The search period was limited to January 1990 to March 2025. The implemented search strategies are shown in Appendix A.

Inclusion criteria were as follows: 1) fertility protection is taken as the main content of this study, which involves defining attributes, concepts, related words or substitutes, pre-factors, and post-effects, evaluation tools, and typical examples, etc. 2) published in English or Chinese; 3) article types included original research, review articles, and non-empirical scholarly papers relevant to concept development. Exclusion criteria were as follows: repeated publication, incomplete, irrelevant, and inaccessible articles.

Based on inclusion and exclusion criteria, two researchers independently screened titles and abstracts to select articles for full-text review. Then they conducted full-text reviews to identify eligible articles. Any disagreements were discussed with another researcher until a consensus was reached.

### 2.3 Data extraction and analysis

Each selected article was carefully read and reviewed. Data were extracted with attention to the author, year of publication, country, study theme, study type, and the role of the article in this concept analysis. In addition, text relevant to the conceptual use of fertility protection was charted from the full texts to support the analysis of the concept’s development, related terms, defining attributes, antecedents, and consequences. Two researchers independently extracted the data into separate Excel spreadsheets. After extraction, cross-checking was performed, and disagreements were resolved through discussion with a third researcher.

The extracted materials were analyzed using thematic content analysis. Concept-related data were compared across studies and grouped according to similarity and meaning. Through iterative discussion and synthesis, themes concerning the concept’s development, related terms, attributes, antecedents, and consequences were identified and organized in Excel to support the final conceptual structure.

## 3 Results

### 3.1 Search outcomes

A total of 18,747 studies were initially retrieved from electronic databases. After searching and removing duplicates, 15,585 articles were obtained, and 180 were screened based on their titles and abstracts. After reviewing the full text, 36 articles were included in the research category. A retrospective search of reference lists identified 4 further relevant articles, bringing the final sample for in-depth analysis to 40 [13,[16], [17], [18], [19], [20], [21], [22], [23], [24], [25], [26], [27], [28], [29], [30], [31], [32], [33], [34], [35], [36], [37], [38], [39], [40], [41], [42], [43], [44], [45], [46], [47], [48], [49], [50], [51], [52], [53], [54]]. Among these, 32 were in English, and 8 were in Chinese. A PRISMA flow diagram showing the search strategy process is presented in Appendix B.

The majority of the literature originated from China (*n* = 14) [17,18,25,26,28,29,31,33,37,38,40,43,44,54], the USA (*n* = 8) [21,32,[34], [35], [36],^42^,^46^,^48^], and the UK (*n* = 5) [16,20,23,41,52], with the remainder from Italy (*n* = 3) [24,27,51], Belgium (*n* = 2) [22,50], France (*n* = 2) [47,49], Germany (*n* = 1) [13], Israel (*n* = 1) [19], Indonesia (*n* = 1) [30], Portugal (*n* = 1) [39], Canada (*n* = 1) [45], and the Netherlands (*n* = 1) [53]. The detailed characteristics of the included studies are shown in Appendix C.

### 3.2 Related terms

From the selected articles, two terms that are used interchangeably with fertility protection concepts were identified: reproductive health and fertility preservation. A detailed comparison of these concepts is presented in Appendix D.

The WHO defines reproductive health as an intact state of physical, psychological, and social adjustment related to the reproductive system and its functions and processes, and not merely the absence of disease or infirmity [55]. As a broader concept, reproductive health includes fertility protection. Reproductive health emphasizes overall health status and quality of life, focusing on the normal functioning of the reproductive system and individuals’ well-being in relation to reproduction. In contrast, fertility protection places greater emphasis on preserving fertility, focusing on how to prevent its decline or loss and how to provide support and assistance to individuals at risk of impaired fertility.

Fertility preservation refers to the storage of reproductive cells, tissues, or embryos through cryopreservation for future use [16]. Fertility preservation is an important technological underpinning of fertility protection in the biomedical field and a concrete means of achieving its goals. Fertility preservation mainly focuses on medical techniques such as oocyte and embryo cryopreservation [17]. Because it is a key component of fertility protection, the terms are often used interchangeably when discussing it. However, fertility protection is broader, encompassing not only fertility preservation techniques but also a wider range of strategies and measures aimed at reducing or preventing fertility damage caused by endogenous and exogenous factors, thereby maintaining individual fertility [18].

### 3.3 Development of the concept

In the late 20th and early 21st centuries, the widespread use of chemotherapy and radiotherapy significantly improved cancer survival [56]. Still, these treatments often caused irreversible reproductive damage, resulting in reduced or lost fertility [19]. This prompted growing concern about how to treat disease while preserving patients’ reproductive potential, leading to the emergence of the concept of fertility protection. In 1994, Gosden et al. [20]. Showed that cryopreserved ovarian tissue could restore fertility after transplantation, opening a new avenue for protecting female fertility. The American Society of Clinical Oncology subsequently defined fertility protection as “the process of preserving germ cells or tissues through medical intervention to maintain the likelihood of future fertility” [21]. With continuing advances in oocyte, ovarian tissue, sperm, and testicular tissue cryopreservation [22,23], the concept gradually expanded beyond cancer care to include individuals at risk of fertility impairment from other diseases or factors, such as endometriosis and autoimmune disorders [24,25]. As its use broadened, fertility protection also evolved into an interdisciplinary field involving reproductive medicine, oncology, and sociology. It now encompasses not only gamete preservation but also preventive interventions and psychosocial support aimed at maintaining, restoring, and enhancing fertility and helping individuals achieve their reproductive goals [13,26,27]. Thus, the concept has shifted from “preservation” to “protection,” while retaining its core concern with balancing medical needs and reproductive rights [17].

### 3.4 Attributes

#### 3.4.1 Prevention-oriented approach

The foundation of fertility protection lies in preventive interventions that employ proactive, forward-looking strategies to safeguard fertility. This protection spans the many phases of a person’s lifecycle, aiming to safeguard fertility from an early age [18]. According to research [28], fertility is influenced by multiple factors, including disease, environment, lifestyle, and iatrogenic exposures. Fertility protection identifies and assesses these risk factors that may impair fertility through a risk stratification system and develops appropriate intervention strategies [26]. From a lifecycle perspective, the focus of risk assessment and intervention varies across life stages [29].

Primary prevention focuses on etiological prevention. During adolescence, reproductive health knowledge should be delivered through schools and community education, including sexual health education and scientific contraceptive methods [30]. Adolescents should also be made aware of the physical and psychological harms of induced abortion and its potential impact on future fertility, to reduce unintended pregnancy [31]. At the same time, healthy lifestyle habits, such as avoiding smoking and excessive alcohol consumption, should be encouraged because unhealthy behaviors may adversely affect future fertility [32]. In addition, post-abortion care should be strengthened by providing women with psychological support, medical follow-up, and recovery guidance [33].

Secondary prevention focuses on early monitoring and intervention. During puberty, attention should be paid to adolescents’ reproductive development, with initial screening conducted through school health examinations. During the reproductive years, regular gynecological and male reproductive health check-ups are needed to detect and treat fertility-related diseases promptly [34]. In addition, uterine procedures, such as abortion and diagnostic curettage, should be performed strictly according to clinical guidelines to avoid complications such as intrauterine adhesions and endometrial damage caused by improper operation [57].

Tertiary prevention aims to minimize fertility damage when it is unavoidable. Typical measures include fertility preservation, such as embryo, oocyte, and sperm cryopreservation, before radiotherapy or chemotherapy [35,36]. In addition, the use of gonadotropin-releasing hormone (GnRH) agonists or antagonists during chemotherapy may reduce fertility damage by suppressing the hypothalamic–pituitary–gonadal (HPG) axis. The three levels of preventive measures discussed above combine to form a comprehensive and systematic system of prevention and control that can minimize the negative effects of various risk factors on fertility.

#### 3.4.2 Promoting the release of fertility potential

Fertility is shaped not only by physiological factors but also by social, economic, and cultural conditions [11]. Although many individuals are biologically capable of childbearing, social pressure, a fast-paced lifestyle, delayed marriage and childbearing, and changing reproductive attitudes often prevent them from fully realizing their fertility potential [37]. Fertility protection, through the dual dimensions of subjective initiative stimulation and objective technical support, inspires and enhances the childbearing intentions and fertility of the target age groups, enabling their full potential fertility to be realized and achieving the reproductive goal. From the perspective of stimulating subjective initiative, increasing childbearing intention is an integral part of promoting the release of fertility potential [38]. On the one hand, age-appropriate childbearing must be promoted through education and publicity to counteract some people’s negative views of childbearing, fully inform people of the benefits of having children for the individual, the family, and society, and dispel any fear of having children brought on by false beliefs [39]. For example, some people have the misconception that “childbearing will seriously affect one’s career development”, believing that once they give birth to children, they will miss the opportunity of promotion in the workplace and be eliminated by society [37,38,58]. However, many women who have given birth still achieve remarkable success in their careers through proper planning and hard work. Ignoring the synchronicity between family growth and personal development, some people also maintain the belief that “only those with perfect conditions are suitable for childbearing," meaning that they can only become parents if they have a stable financial foundation (such as a house, car, and a high income) and a perfect marital status [39]. On the other hand, to address people’s reluctance to have children due to financial burdens, the government and society should implement several supportive policies to reduce the financial strain on childbearing families [28,40]. These policies should include maternity subsidies, education subsidies, tax breaks, and more [28,41]. This will increase people’s intention to give birth and proactively unlock their fertility potential [28]. In terms of objective technical support, assisted reproductive technologies (ART) provide an important means of promoting the release of fertility potential [42]. For those who have difficulties in having children due to physiological reasons, ART, such as in vitro fertilization (IVF) and artificial insemination, can effectively help them realize their desire to have children.

#### 3.4.3 Orientation towards the goal of building a birth-friendly society

A birth-friendly society is a favorable social state in which all aspects of society respect and support procreation [43]. This social pattern is not only concerned with numerical changes in birth rates but also focuses on the formation of a benign operating mechanism that respects and supports procreation in the dimensions of physiological guarantee, psychological support, social inclusion, and institutional innovation. The introduction of the concept of a birth-friendly society has stimulated the emergence of new connotations of fertility protection [43,44]. Oriented towards the goal of building a birth-friendly society, fertility protection is no longer confined to maintaining individual fertility. Still, it emphasizes the creation of an integrated environment that supports reproduction through the synergistic effects of policy, culture, the economy, and social services [37,44]. This orientation has led healthcare professionals, policymakers, and researchers to focus on the social influences on fertility and to promote synergistic optimization of medical interventions and social support. The goal of a birth-friendly society can only be fully achieved when everyone’s fertility is fully protected, that is, when there is adequate health maintenance at the physiological level, effective counseling and support at the psychological level, and perfect protection of rights and interests at the social level.

### 3.5 Antecedents

#### 3.5.1 Physiological factors

Age is the most important physiological factor affecting fertility. According to the research, women are most fertile between 23 and 32 years of age, after which fertility gradually declines because ovarian function deteriorates and both oocyte number and quality decrease [59]. For men, sperm count, viability, and morphology also decline after age 35 [60]. These changes reduce the chance of conception, impair embryo implantation and development, and increase the risks of miscarriage and fetal congenital disabilities. Abnormal body mass index (BMI), whether high or low, is another important factor. Women with a high BMI often have insulin resistance, which causes ovulation disorders and affects fertility [61]. In men, a high BMI is associated with increased reactive oxygen species in seminal plasma, reduced glutathione peroxidase activity, and higher rates of early sperm apoptosis [62]. Low BMI may also impair female fertility by causing hypothalamic amenorrhea through insufficient leptin levels and disrupted GnRH pulse secretion [63].

#### 3.5.2 Pathological factors

Reproductive dysfunction in females is frequently associated with gynecological pathologies such as ovarian cysts, polycystic ovary syndrome (PCOS), tubal obstruction, and endometriosis [64]. These conditions may adversely affect critical reproductive processes, including oogenesis, ovum release, gamete transport, and embryo implantation, ultimately contributing to diminished fertility. Similarly, male reproductive capacity can be compromised by urogenital disorders such as orchitis, epididymitis, and varicocele, which may impair spermatogenesis and sperm quality [65]. Furthermore, systemic endocrine-metabolic pathologies, particularly thyroid dysfunction and diabetes mellitus, may disrupt sex hormone synthesis through metabolic imbalance, impair germ cell maturation in both sexes, and ultimately contribute to declining fertility [66].

#### 3.5.3 Iatrogenic factors

Iatrogenic factors include medical interventions such as surgery, chemotherapy, radiotherapy, immunotherapy, and abortion. Some surgical procedures may impair fertility; for example, ovarian cyst removal may disrupt ovarian blood supply and reduce follicle number [45], while radical prostatectomy may damage the nerves controlling ejaculation and lead to retrograde ejaculation or erectile dysfunction [46]. Chemotherapeutic drugs such as cyclophosphamide have direct toxic effects on germ cells. For women, it can damage primordial follicles in the ovaries, leading to decreased ovarian reserve function; for men, it can inhibit testicular spermatogenesis, reducing sperm count and vigor [47]. Radiotherapy causes radiation damage to the reproductive organs, damaging the DNA of reproductive cells and leading to chromosomal aberrations [48]. Immune checkpoint inhibitors may cause reproductive organ damage by overactivating the immune system [49]. For example, PD-1/PD-L1 inhibitors may cause ovarian damage through T-cell-mediated autoimmune attack, whereas CTLA-4 inhibitors may impair male fertility by disrupting testosterone production and spermatogenesis [50]. In addition, abortion may reduce fertility [67]. Irregular practices or postoperative infections may lead to endometrial damage, intrauterine adhesions, or chronic inflammation, while multiple abortions also further increase the risk of complications such as uterine adhesions, cervical laxity, and endometrial damage.

#### 3.5.4 Psychological factors

Prolonged mental stress and depression trigger the hypothalamic-pituitary-adrenal (HPA) axis, which raises cortisol levels. These elevated cortisol levels disrupt the normal functioning of the hypothalamic-pituitary-ovarian (HPO) and hypothalamic-pituitary-testicular (HPT) axes, which, in turn, influence gonadotropin secretion, resulting in abnormal spermatogenesis and ovulation disorders [51,68]. Anxiety may provoke difficulties such as erectile dysfunction and premature ejaculation in males and diminished libido and vaginal cramps in women. These sexual dysfunctions can have a detrimental impact on fertility by directly reducing the chances of conception [69].

#### 3.5.5 Environment and lifestyle

Long-term exposure to environmental pollutants such as polychlorinated biphenyls, organochlorine pesticides, and phthalates may disrupt endocrine function and impair reproductive cell development [70]. Exposure to harmful substances in the workplace, including heavy metals and chemicals, may also damage the reproductive system. In addition, unhealthy lifestyle habits, such as chronic sleep deprivation, physical inactivity, smoking, alcohol misuse, and excessive coffee consumption, can adversely affect reproductive health and reduce fertility [7].

#### 3.5.6 Social factors

From a socio-economic perspective, many families are reluctant to have children due to rising living costs, including housing, education, and healthcare [71]. To preserve the standard of living for current family members, people sometimes postpone or have fewer children when economic pressures are high. Inadequate social support can further constrain fertility, whereas sound childcare policies and adequate childcare services may reduce the burden of childbirth and child-rearing and strengthen childbearing intentions [40]. In terms of education level, those with higher education are more likely to focus on personal career development, are more inclined to pursue financial independence, and are relatively conservative in their birth planning [58]. In terms of social attitudes, people are becoming more conscious of their self-realization and quality of life, whereas the old ideas of “more children, more happiness,” and “raising children for old age” are progressively disappearing [58,71]. The rise of individualism and liberal thinking has made some people more inclined to choose the small-family model or even the Dink lifestyle, and they no longer regard procreation as an inevitable life choice [28,40]. This weakens the desire to have children, which in turn affects fertility.

### 3.6 Consequences

#### 3.6.1 Individual-related consequences

Reproductive decision-making among people of childbearing age typically involves a psychological process from childbearing motivation to childbearing desire, childbearing intention, childbearing planning or decision-making, and finally childbearing behavior [72,73]. This process begins with the emergence of procreative motivation, which serves as the internal driving force for subsequent decision-making [74]. At the unconscious level, the reproductive subject is inclined to be fertile or infertile, and this inclination further influences whether they develop childbearing desire at the conscious level. When the fertility desire is formed, the subject of procreation will form a clear childbearing intention on this basis, and finally decide to procreate by considering the external environmental factors [52]. The outcome of this decision will directly affect whether to converge on childbearing or avoidance behavior. Fertility protection may strengthen childbearing motivation, desire, and intention, thereby facilitating reproductive decision-making and ultimately promoting actual childbearing behavior and the achievement of reproductive goals [53,54].

#### 3.6.2 Family-related consequences

Fertility protection can help families achieve their desired parenthood and support a healthy family structure. Families that can conceive successfully and raise healthy children are more likely to maintain harmonious spousal and parent-child relationships [75]. Fertility protection may also reduce the burden of childbearing and childrearing and enhance family well-being. Through financial support and policy protection, it can lower childcare-related expenses, allow families to allocate more resources to other needs, and improve overall quality of life [41]. In addition, fertility protection may reduce hidden costs and family stress [76]. While traditional concepts of fertility tend to be about letting nature take its course, fertility protection makes people realize that fertility is a resource that needs to be actively managed and protected. Encouraging active fertility planning and management can help prevent the high medical expenses and psychological distress associated with infertility or prolonged difficulty conceiving, thereby easing both financial and emotional burdens on families.

#### 3.6.3 Social-related consequences

Fertility protection may increase childbearing intention and fertility, thereby raising the fertility rate, optimizing the demographic structure, and alleviating pressures associated with population aging [77]. It may also promote gender equality by protecting women’s reproductive rights and health and reducing inequalities related to childbearing. For example, birth-friendly policies such as flexible working arrangements and adequate maternity and parental leave can help women better balance work and family responsibilities, reduce childbirth-related stress and conflict, and improve their social status [78]. In turn, greater gender equality may further strengthen childbearing intention and fertility, creating a positive cycle. Fertility protection may also benefit economic development. Higher fertility can expand demand in childbirth-related sectors, including healthcare, early childhood education, and maternal and child products, thereby generating employment opportunities and supporting related industries [79]. In the longer term, growth in the newborn population may provide human resources for economic development and contribute to technological innovation and social progress [28].

### 3.7 Model case

Mary, a 28-year-old unmarried and childless woman, was diagnosed with early-stage breast cancer requiring chemotherapy. Her physician informed her that chemotherapy could cause irreversible ovarian damage and recommended fertility protection, such as ovarian tissue cryopreservation (based on preventive interventions). After receiving detailed counseling from the nurse and consulting the reproductive medicine department, Mary chose ovarian tissue cryopreservation before treatment. Five years later, after her condition had stabilized, she was married and planning a pregnancy, but had difficulty conceiving naturally. She therefore underwent ovarian tissue transplantation combined with ART. Although she was initially concerned about the cost, these services were covered by local health insurance, which enabled her to achieve a successful pregnancy and delivery (promoting the release of fertility potential). In addition, her employer provided flexible working hours and job retention during treatment and maternity leave (an orientation towards the goal of building a birth-friendly society).

### 3.8 Definition of fertility protection

Fertility protection refers to the maintenance or preservation of an individual’s fertility through preventive interventions, while at the same time promoting the full exertion of their fertility potential through a combination of medical technology, policy, economic, and socio-cultural means, thereby achieving the ultimate goal of a birth-friendly society. A visual overview of the conceptual structure of fertility protection is shown in Appendix E.

## 4 Discussion

Analysis of the literature reveals that the conceptual evolution of fertility protection reflects the transition from the medical model to an integrated biopsychosocial model. Traditionally, fertility protection has been equated with fertility preservation technologies for cancer patients [13]. However, as global fertility rates decline, trends of delayed marriage and childbirth increase, and lifestyle changes and economic pressures on childbearing families intensify [2,3,40], the limitations of a solely biomedical approach have become increasingly evident. Fertility protection now encompasses life-course prevention strategies, psychosocial support, and societal empowerment. This study found that the core attributes of fertility protection include not only preventive interventions from a traditional medical perspective but also encompass the goal of promoting the release of fertility potential and building a birth-friendly society through policy and cultural reforms. These three attributes are interdependent and dynamically interactive. For example, preventive interventions, such as ovarian tissue cryopreservation in cancer patients, directly protect physiological fertility, but their effectiveness often depends on societal support, such as health insurance coverage. At the same time, a birth-friendly society, including policies such as flexible parental leave, can strengthen childbearing intention and indirectly promote the uptake of preventive interventions.

### 4.1 Practice implications of preventive interventions

Based on preventive interventions, fertility protection should encompass primary, secondary, and tertiary prevention, rather than being limited to a small group of high-risk populations. In primary prevention, nurses can enhance reproductive health and contraception knowledge among adolescents and reproductive-age individuals through school and community health education [30,31], thereby reducing preventable fertility damage. Secondary prevention emphasizes regular screening and early intervention, such as regular gynecological check-ups and male reproductive health check-ups, and standardized perioperative procedures, to minimize the impact of diseases and iatrogenic factors on fertility [34,57]. Tertiary prevention focuses on high-risk or diagnosed patients, ensuring timely communication and referral for fertility assessments and preservation options before treatments with potential reproductive toxicity, such as cancer therapies [35,36].

### 4.2 Multidimensional strategies to promote the release of fertility potential

Promoting the release of fertility potential means that fertility protection involves not only maintaining biological reproductive capacity but also supporting individuals in achieving their reproductive goals when desired. At the subjective level, psychosocial support is needed to reduce anxiety and misconceptions about childbearing and to strengthen childbearing motivation and intention among people of reproductive age [38,39]. At the objective level, ART should be effectively utilized to help individuals with physiological barriers to conception achieve their reproductive aspirations [42], while providing comprehensive information and decision-making support. In addition, structural factors such as housing, education, and childcare costs may suppress childbearing behavior, making economic and social policies, including subsidies, tax incentives, and childcare services, important external conditions for releasing fertility potential [28,40]. These multidimensional strategies work synergistically, with individual-level support, technology application, and policy safeguards collectively driving the comprehensive release of fertility potential.

### 4.3 Collaborative pathways toward a birth-friendly society

An orientation toward the goal of building a birth-friendly society indicates that fertility protection has transcended a singular clinical issue and now involves a broader social support environment [37,44]. At the institutional level, establishing systematic protocols for fertility risk assessment and counseling, optimizing referral pathways, and integrating fertility protection into quality-of-care metrics are essential steps. Nurses can play an advocacy and driving role within multidisciplinary teams and quality improvement projects. At the policy and societal levels, expanding coverage for fertility preservation technologies and assisted reproductive services, improving maternity and parental leave systems, protecting reproductive labor rights, and developing accessible childcare and family-friendly community environments all contribute to reducing the burden of childbearing and enhancing the birth-friendliness of families and society.

### 4.4 Establishing a unified framework for interdisciplinary collaboration

Through concept analysis, the three interrelated defining attributes, six categories of antecedents, and three key outcomes of fertility protection have been clarified, providing a unified framework for interdisciplinary collaboration among oncology, reproductive medicine, nursing, psychology, and public policy. This framework helps define the roles and responsibilities of each discipline in pursuit of common goals. For example, oncology and reproductive medicine specialists can focus on developing individualized fertility protection strategies and reducing iatrogenic damage; nurses can prioritize patient education, psychosocial support, and care coordination; public health professionals can design interventions targeting modifiable risk factors in at-risk populations; and policymakers can use this framework to develop regulations and funding mechanisms that align with the goal of a “birth-friendly society.” This interdisciplinary collaboration can drive the comprehensive implementation of fertility protection, thereby promoting reproductive health and sustainable development.

### 4.5 Limitations

The literature analysis in this study is limited to English and Chinese sources, potentially overlooking important research and practical outcomes from other linguistic and cultural contexts. Furthermore, although the conceptual framework in this study has broad applicability, its specific relevance and effectiveness in different cultural contexts, healthcare systems, and socioeconomic conditions require further empirical validation.

## 5 Conclusions

Fertility protection is a multidimensional concept centered on maintaining or preserving fertility through preventive interventions while promoting the full release of fertility potential through medical, psychosocial, policy, economic, and socio-cultural support. This analysis shows that fertility protection is characterized by three core attributes: a prevention-based approach, promotion of fertility potential, and an orientation toward a birth-friendly society. It also identifies six categories of antecedents and consequences across individual, family, and societal domains. By clarifying these conceptual components, this study provides a foundation for more consistent use of the term in nursing, reproductive medicine, public health, and policy research. Multidisciplinary collaboration and the conduct of comprehensive fertility protection research projects should be the main goals of future fertility protection research.

## Data availability statement

Data sharing is not applicable to this article as no datasets were generated or analyzed during the current study.

## CRediT authorship contribution statement

**Yaxin Chen:** Conceptualization, Methodology, Validation, Formal analysis, Investigation, Data curation, Writing - original draft, Writing - review & editing, Project administration. **Xin Qi:** Conceptualization, Methodology, Validation, Formal analysis, Investigation, Resources, Data curation, Writing - review & editing. **Mengyuan Yang:** Conceptualization, Methodology, Validation, Formal analysis, Investigation, Resources, Data curation, Writing - review & editing. **Jing Li:** Conceptualization, Methodology, Validation, Formal analysis, Investigation, Resources, Writing - review & editing. **Lei Wu:** Conceptualization, Methodology, Validation, Formal analysis, Investigation, Resources, Writing - review & editing. **Yaya Zhang:** Conceptualization, Methodology, Validation, Formal analysis, Investigation, Resources, Writing - review & editing. **Zhentao Yang:** Conceptualization, Methodology, Validation, Formal analysis, Investigation, Resources, Writing - review & editing. **Ruirui Wang:** Conceptualization, Methodology, Validation, Formal analysis, Investigation, Resources, Writing - review & editing. **Yanhong Wang:** Conceptualization, Methodology, Validation, Formal analysis, Writing - review & editing, Supervision, Project administration.

## Funding

This study was supported by the project “Study on the Joint Developmental Trajectories of Sleep Quality and Physical Activity and Multidimensional Interventions in Pregnant Women” supported by the Science and Technology Plan Project of Chengguan District, Lanzhou City, Gansu Province (grant number: 2023SHFZ0003).

## Declaration of competing interest

The authors have declared no conflict of interest.
