# Supplementary material for: Fertility protection in the reproductive-age population: A evolutionary concept analysis
Source: Int J Nurs Sci. 2026 Apr 22;13(5):527–34. doi: 10.1016/j.ijnss.2026.04.011 (PMC13648180; doi:10.1016/j.ijnss.2026.04.011)
Supplement: Supplementary file 1 [file mmc1.docx]

Appendix A The detailed search strategies of each database.

| Databases | Retrieval strategy |
| --- | --- |
| PubMed | ("fertility protection"[Title/Abstract]) OR ("fertility preservation"[Title/Abstract] NOT "cryopreservation"[Title/Abstract]) OR ("impaired fertility"[Title/Abstract]) OR ("decreased fertility"[Title/Abstract]) OR ("reduced fertility"[Title/Abstract]) |
| Web of Science | TS=("fertility protection") OR TS=("fertility preservation" NOT "cryopreservation") OR TS=("impaired fertility") OR TS=("decreased fertility") OR TS=("reduced fertility") |
| CNKI | (SU="生育力保护") OR (SU="生育力保存") OR (SU="生育力保留") OR (SU="生育能力保护") OR (SU="生育能力保存") OR (SU="生育能力保留") OR (SU="生殖力保护") OR (SU="生殖力保存") OR (SU="生殖力保留") |
| CBM | ("生育力保护" [常用字段:智能]) OR ("生育力保存" [常用字段:智能]) OR ("生育力保留" [常用字段:智能]) OR ("生育能力保护" [常用字段:智能]) OR ("生育能力保存" [常用字段:智能]) OR ("生育能力保留" [常用字段:智能]) OR ("生殖力保护" [常用字段:智能]) OR ("生殖力保存" [常用字段:智能]) OR ("生殖力保留" [常用字段:智能]) |

*Note:* CNKI = China National Knowledge Infrastructure. CBM: China Biomedical Literature Database.

Records retained for extraction

(*n* = 40)

Additional studies identified through reference list searching (*n* = 4)

Records identified from databases (*n* = 18,747):

PubMed (*n* = 8,043); Web of science (*n* = 8,569); China National Knowledge Infrastructure (*n* = 1,305); China Biomedical Literature Database (*n* = 830)

Records removed before screening:

Duplicate records removed (*n* = 3,162)

Records screened

(*n* = 15,585)

**Identification**

Reports assessed for eligibility

(*n* = 180)

Reports excluded (*n* = 144)

- Authors did not describe at least one fertility protection attribute, antecedent, or consequence of fertility protection (*n* = 84)
- Article types did not meet the inclusion criteria (*n* = 43)
- Inaccessible articles (*n* = 11)
- No English or Chinese (*n* = 6)

Records excluded by reading titles and abstracts (*n* = 15,405)

Studies included from database search

(*n* = 36)

**Screening**

**Included**

Appendix B Literature search and selection flowchart.

Appendix C Characteristics of the included studies.

| Study | Country | Study theme | Study type | Role in this concept analysis |
| --- | --- | --- | --- | --- |
| Bundschu, et al., 2025 [13] | Germany | Future perspectives of fertility protection. | Narrative review | Clarified concept evolution and future scope. |
| Gosden, 2009 [16] | UK | Definition, history, and prospect of fertility preservation. | Narrative review | Supported related term comparison and historical development. |
| Xu & Yang, 2023 [17] | China | Evolution and expansion of the concept of fertility protection. | Theoretical paper | Supported concept evolution from preservation to protection. |
| Xu & Yang, 2024 [18] | China | Transformation from fertility technology protection to. comprehensive protection. | Commentary | Supported expansion of conceptual attributes. |
| Meirow & Nugent, 2001 [19] | Israel | Effects of radiotherapy and chemotherapy on female reproduction. | Narrative review | Supported iatrogenic antecedents. |
| Gosden et al., 1994 [20] | UK | Restoration of fertility after ovarian tissue autograft. | Experimental study | Supported early historical basis of fertility protection. |
| Loren et al., 2013 [21] | USA | Fertility preservation for patients with cancer. | Guideline | Supported medical/technical dimension and tertiary prevention. |
| Giudice et al., 2019 [22] | Belgium | Male fertility preservation in high-risk populations. | Narrative review | Supported male fertility protection strategies. |
| Anderson et al, 2020 [23] | UK | Female fertility preservation. | Guideline | Supported female fertility protection strategies. |
| La Marca et al., 2025 [24] | Italy | Fertility preservation in women with endometriosis. | Narrative review | Supported extension beyond oncology. |
| Qiao & Li, 2014 [25] | China | Challenges and opportunities in fertility preservation. | Commentary | Supported conceptual expansion and interdisciplinary relevance. |
| Huang, 2016 [26] | China | Fertility protection overview. | Narrative review | Supported broad conceptual framing. |
| Montano et al., 2022 [27] | Italy | Lifestyle intervention and semen quality. | Randomized controlled trial | Supported preventive interventions and lifestyle protection. |
| Zhang et al., 2023 [28] | China | Family fertility support policies and fertility improvement. | Mixed methods research | Supported social-policy pathways for releasing fertility potential. |
| Lin & Zhu, 2024 [29] | China | Three levels of prevention for female fertility protection. | Narrative review | Supported preventive interventions across the life course. |
| Utami et al., 2024 [30] | Indonesia | School-based sexual and reproductive health education. | Narrative review | Supported primary prevention. |
| Wang et al., 2020 [31] | China | Prevention of abortion and adolescent fertility protection. | Commentary | Supported primary prevention and adolescent fertility protection. |
| Penzias A, 2022 [32] | USA | Natural fertility optimization. | Committee opinion | Supported lifestyle-related preventive interventions. |
| Wang et al., 2024 [33] | China | Post-abortion care and women’s fertility protection. | Systematic review and meta-analysis | Supported post-abortion fertility protection and primary/secondary prevention. |
| Warner et al., 2015 [34] | USA | Detection, prevention, and management of infertility. | Commentary | Supported secondary prevention and public health perspective. |
| Christianson & Oktay, 2019 [35] | USA | Fertility-preservation surgery. | Narrative review | Supported tertiary prevention and medical intervention. |
| Holoch & Wald, 2011 [36] | USA | Male fertility preservation options. | Narrative review | Supported male fertility preservation pathways. |
| Liu et al., 2020 [37] | China | Population fertility and fertility protection. | Theoretical paper | Supported the social dimension of fertility protection. |
| Yang et al., 2023 [38] | China | Third-child fertility intention and socioeconomic factors. | Cross-sectional study | Supported fertility intention and release of fertility potential. |
| Martins et al., 2024 [39] | Portugal | Fertility education and fertility literacy tools. | Recommendation paper | Supported subjective motivation and fertility awareness. |
| Li et al., 2024 [40] | China | Work-family conflict and women’s fertility intention. | Cross-sectional study | Supported social antecedents affecting fertility potential. |
| Laroque & Salanié, 2014 [41] | UK | Response of fertility to financial incentives. | Mixed methods research | Supported economic support and family-related consequences. |
| Adamson et al., 2023 [42] | USA | Global fertility care with assisted reproductive technologies. | Narrative review | Supported technical support for releasing fertility potential. |
| Ye, 2023 [43] | China | Legal framework for building a birth-friendly society. | Theoretical pape | Supported a birth-friendly society orientation. |
| Yu & Liu, 2023 [44] | China | Internal logic and practice of a fertility-friendly society. | Theoretical paper | Supported a birth-friendly society orientation. |
| Russo et al., 2025 [45] | Canada | Ovarian cystectomy and oocyte retrieval for fertility preservation. | Commentary | Supported iatrogenic antecedents and fertility-preserving intervention. |
| Sigman, 2021 [46] | USA | Iatrogenic male infertility due to treatment. | Commentary | Supported iatrogenic antecedents. |
| Rives et al., 2022 [47] | France | Fertility preservation for patients with cancer. | Guideline | Supported tertiary prevention and clinical pathways. |
| Bates et al., 2016 [48] | USA | Fertility and cancer treatment. | Narrative review | Supported cancer-related iatrogenic fertility impairment. |
| Leroy et al., 2015 [49] | France | Immunosuppressive drugs and fertility. | **Narrative review** | Supported drug-related iatrogenic antecedents. |
| Ntemou et al., 2024 [50] | Belgium | Immune checkpoint inhibitors and male fertility. | **Narrative review** | Supported novel treatment-related iatrogenic antecedents. |
| Palomba et al., 2018 [51] | Italy | Stress, quality of life, and female fertility. | **Narrative review** | Supported psychological antecedents. |
| Harper et al., 2021 [52] | UK | Fertility education initiative and awareness. | Commentary | Supported fertility awareness and motivational mechanisms. |
| Fauser et al., 2024 [53] | Netherlands | Declining global fertility and family planning/family building. | Expert consensus statement | Supported broader consequences and social relevance. |
| Ren et al., 2024 [54] | China | Fertility intention among young people in China. | Systematic review and meta-analysis | Supported individual consequences and fertility intention formation. |

Appendix D Distinguishing fertility protection from related terms.

| Dimension | Fertility protection | Reproductive health | Fertility preservation |
| --- | --- | --- | --- |
| Core Focus | Maintain and enhance fertility potential through integrated measures | Overall well-being of the reproductive system and processes | Storing reproductive materials for future use |
| **Target Population** | All individuals with fertility potential | All individuals across the lifecycle (adolescents, childbearing-age individuals, older adults) | Patients at imminent risk of iatrogenic or disease-related infertility |
| **Temporal Perspective** | Lifespan-oriented, preventive | Continuous, holistic. | Pre-treatment, reactive |
| **Scope of Interventions** | Medical, educational, policy, economic, socio-cultural. | Healthcare services, education, rights, counseling | Technical (cryopreservation, surgery) |
| **Primary Disciplinary Field** | Multidisciplinary (nursing, reproductive medicine, oncology, public health, psychology, and sociology) | Public health and medicine | Reproductive medicine and oncology |

**Social factors** (e.g., socio-economic factors, social support, social attitudes)

**Environment and lifestyle** (e.g., environmental pollutants, heavy metals, chemicals, and unhealthy lifestyle habits)

**Psychological factors** (e.g., Prolonged mental stress, depression, and anxiety)

**Iatrogenic factors** (e.g., surgery, chemotherapy, radiotherapy, immunotherapy, and abortion)

**Pathological factors** (e.g., gynecological pathologies, urogenital disorders, systemic endocrine-metabolic and pathologies)

**Antecedents**

**Attributes**

**Consequences**

**Physiological factors** (e.g., age and body mass index)

**Prevention-oriented approach:**

Whole life-cycle protection concept, three levels of preventive measures.

**Individual-related consequences:**

Enhancing childbearing motivation, childbearing desire, and childbearing intention among childbearing-age populations, ultimately translating fertility intentions into actual reproductive behaviors.

**Promoting the release of fertility potential:**

The dual dimensions of subjective initiative stimulation and objective technical support.

**Orientation towards the goal of building a birth-friendly society**

**Family-related consequences:**

Facilitating the realization of fertility goals, fostering healthy family structures, alleviating childcare burdens while improving family well-being, and reducing hidden costs associated with procreation.

**Social-related consequences:**

Elevating population fertility rates, demographic structural optimization and mitigation of population aging pressures, reduction of gender inequality caused by reproductive issues, and stimulation of sustainable economic development.

Appendix E Conceptual structure of fertility protection.
