# Supplementary material for: Fertility protection in the reproductive-age population: A evolutionary concept analysis
Source: Int J Nurs Sci. 2026 Apr 22;13(5):527–34. doi: 10.1016/j.ijnss.2026.04.011 (PMC13648180; doi:10.1016/j.ijnss.2026.04.011)
Supplement: Supplementary file 2 [file mmc2.docx]

**育龄期人群生育力保护的演化概念分析**

陈雅馨，漆芯，杨梦圆，李晶，武蕾，张亚亚，杨镇滔，王瑞瑞，王艳红

【**摘要**】

**目的** 对育龄期人群的生育力保护进行概念分析，并阐明其定义。

**方法** 采用Rodgers演化概念分析法，系统检索PubMed、Web of Science、中国知网和中国生物医学文献数据库中有关生育力保护的相关文献，检索时限为1990 年1月至2025年3月。2名研究者根据提前制订的纳入与排除标准进行文献筛选。提取识别生育力保护的替代术语、相关概念、属性特征、前因及后果，并采用主题内容分析法对提取的资料进行综合分析。

**结果** 共纳入40篇文献。生育力保护的概念属性包括预防性干预措施、促进生育潜能释放，以及以构建生育友好型社会为目标导向。其前因被归纳为6类：生理因素、病理因素、医源性因素、心理因素、环境与生活方式因素以及社会因素。其后果则系统归纳为3个层面：个体层面、家庭层面和社会层面。最后，生育力保护被定义为以预防性干预措施为基础，维护或保存个体的生育力，同时通过医疗技术、政策、经济、社会文化等综合手段，促进其生育力潜能的充分发挥，从而实现生育友好型社会的最终目标。

**结论** 生育力保护是一个涉及多学科复杂领域的概念，该研究明确了其概念属性，并为护理学、生殖医学、公共卫生及政策研究之间的有效协作与沟通提供了共同的理论参考基础。

【**关键词**】概念分析；定义；演化概念分析；生育力保护

**通信作者：**王艳红，E-mail：yanhongwang@lzu.edu.cn
